# Visual Acuity Measures Do Not Reliably Detect Childhood Refractive Error - an Epidemiological Study

**DOI:** 10.1371/journal.pone.0034441

**Published:** 2012-03-28

**Authors:** Lisa O'Donoghue, Alicja R. Rudnicka, Julie F. McClelland, Nicola S. Logan, Kathryn J. Saunders

**Affiliations:** 1 School of Biomedical Sciences, University of Ulster, Coleraine, N. Ireland, United Kingdom; 2 Division of Population Health Sciences and Education, St George's, University of London, London, United Kingdom; 3 School of Life and Health Sciences, Aston University, Birmingham, United Kingdom; University of Ottawa, Canada

## Abstract

**Purpose:**

To investigate the utility of uncorrected visual acuity measures in screening for refractive error in white school children aged 6-7-years and 12-13-years.

**Methods:**

The Northern Ireland Childhood Errors of Refraction (NICER) study used a stratified random cluster design to recruit children from schools in Northern Ireland. Detailed eye examinations included assessment of logMAR visual acuity and cycloplegic autorefraction. Spherical equivalent refractive data from the right eye were used to classify significant refractive error as myopia of at least 1DS, hyperopia as greater than +3.50DS and astigmatism as greater than 1.50DC, whether it occurred in isolation or in association with myopia or hyperopia.

**Results:**

Results are presented from 661 white 12-13-year-old and 392 white 6-7-year-old school-children. Using a cut-off of uncorrected visual acuity poorer than 0.20 logMAR to detect significant refractive error gave a sensitivity of 50% and specificity of 92% in 6-7-year-olds and 73% and 93% respectively in 12-13-year-olds. In 12-13-year-old children a cut-off of poorer than 0.20 logMAR had a sensitivity of 92% and a specificity of 91% in detecting myopia and a sensitivity of 41% and a specificity of 84% in detecting hyperopia.

**Conclusions:**

Vision screening using logMAR acuity can reliably detect myopia, but not hyperopia or astigmatism in school-age children. Providers of vision screening programs should be cognisant that where detection of uncorrected hyperopic and/or astigmatic refractive error is an aspiration, current UK protocols will not effectively deliver.

## Introduction

Whilst the main target condition of childhood vision screening programs is amblyopia, the UK National Screening Committee (NSC) vision screening program includes strabismus and uncorrected refractive error as target conditions [1], [2]. Most programs, including those in the United Kingdom, rely on the assessment of uncorrected distance visual acuity (VA) to identify visual impairment [1] and the use of near vision testing is not currently recommended [3]. The UK National Screening Committee guidance supports a single vision screening intervention at 4–5 years using logMAR measures of monocular acuity [2]–[3]. A monocular acuity of poorer than 0.20 logMAR indicates failure under the current criteria [3], [4]. Although uncorrected distance VA measures screen reliably for childhood myopia [5], [6] there is evidence that they cannot be used to screen reliably for either hyperopia or astigmatism [5]. The Northern Ireland Childhood Errors of Refraction (NICER) study, an epidemiological survey of the refractive status in childhood in the UK has reported a high prevalence of both astigmatism [7] and hyperopia [8]. Hyperopia is a known risk factor for the development of strabismus and amblyopia [9], [10], and there is a growing body of evidence to suggest that uncorrected hyperopia may be linked to poorer academic performance [11], [12] and deficits in visuocognitive and visuomotor measures [13].

The aim of this paper is to examine whether the use of uncorrected distance VA is an appropriate screening tool in identifying refractive error in populations with a high prevalence of hyperopia and/or astigmatism.

## Methods

### Ethics Statement

This study was approved by the University of Ulster Research Ethics Committee and the conduct of the study adhered to the tenets of the Declaration of Helsinki. After an explanation of the nature and possible consequences of the study, written consent was obtained from the parents/guardian of all children prior to the examination. The 12-13-year-old children also gave written consent, while verbal assent was obtained from the 6-7-year-old children.

### Subjects

The Northern Ireland Childhood Errors of Refraction (NICER) study is a population-based survey of school children living in Northern Ireland. The study methods have previously been described in detail [14]. In brief, stratified random sampling of schools from geographic areas characteristic of Northern Ireland was employed to obtain a representation of schools and children from urban/rural and deprived/non-deprived areas. Within individual schools, all children in one or more classes were invited to participate. Potential participants were aged 6-7-years and 12-13-years. The protocol for data collection included measurement of logMAR monocular distance VA (uncorrected and with spectacles if worn) using a Windows-based computerised test chart (Test Chart 2000, Thomson Software Solutions, Hatfield, UK) at a distance of at least 3 m. A forced-choice procedure and by-letter scoring [15] was used to determine VA. Cycloplegic autorefraction (1% cyclopentolate hydrochloride, Minims® single dose, Chauvin Pharmaceuticals, Romford, UK) using a binocular open-field autorefractor (Shin-Nippon SRW-5000, Tokyo, Japan) was employed. At least five measurements were taken, with the representative value as determined by the instrument used in subsequent analyses. This autorefractor permits reliable measures of both the spherical and cylindrical (±0.24D SD) components of refraction [16]. Participants were tested within school premises during the school day, between May 2006 and March 2008.

### Data analysis and definitions

The spherical equivalent refraction (sphere +½ cylinder, SER) has been used to classify myopia as at least −1.00DS or more myopia [17], hyperopia as >+3.50DS and astigmatism as >1.50DC whether or not it occurs in association with myopia or hyperopia [18]. Significant refractive error is defined as myopia or hyperopia, and/or astigmatism.

### Statistical analysis

All statistical analyses were carried out using Intercooled Stata 9.2 (StataCorp, Texas, USA). As uncorrected VA is correlated between the right and left eyes (Spearman correlation 0.39, p<0.001) only data from the right eye are presented. As VA data are not normally distributed, median and inter-quartile ranges have been used to describe the distribution of VA and the Wilcoxon rank sum has been employed to study age group differences in VA. Prevalence rates of significant refractive error using right eye data, with 95% confidence intervals, have been adjusted for the cluster design. Chi-squared tests have been used to examine age group differences in the prevalence of significant refractive error, myopia, hyperopia and astigmatism. Sensitivity and specificity values and Receiver Operating Curves were examined to ascertain the best cut-off point (taken as the point closest to the top left-hand corner of the ROC curve) of uncorrected logMAR acuity to detect significant refractive error. The sensitivity and specificity of uncorrected VA of poorer than 0.20logMAR in detecting significant refractive error, myopia, hyperopia and astigmatism is also presented as this criterion is currently recommended by the UK National Screening Committee [3]. Throughout, results are considered statistically significant if p<0.05.

## Results

Of the children invited to participate in the study, parental consent was obtained from 65% of 12-13-year-olds and 62% of 6-7-year-olds. Reflective of the Northern Irish population, 98.7% of participants were white and this report presents data from 661 white children aged 12-13-years (50.5% male) and 392 white children aged 6-7-years (49.5% male). The mean ages of the two groups studied were 13.1 years (range 12.1–14.1 years) and 7.1 years (range 6.3–7.8 years) respectively.

As expected, VA data in both 6-7-year olds and 12-13-year olds were skewed. There was a statistically significant difference in uncorrected VA between 6-7-year-old (median 0.10, IQR 0.04 to 0.14) and 12-13-year-old children (median 0.00, IQR −0.06 to 0.12) (p<0.001).

Table 1 presents data on the prevalence of significant refractive error, myopia, hyperopia and astigmatism. Whilst there was no statistically significant difference in the prevalence of hyperopia and astigmatism between the two age groups (p both>0.51) there was a significant difference in the prevalence of significant refractive error and myopia (p both<0.001).

**Table 1:** The prevalence of significant refractive error, myopia, hyperopia and astigmatism.

		Prevalence (%, 95% CIs)			
Age-Group (yrs)		Significant refractive error	Myopia≤−1DS	Hyperopia>+3.50DS	Astigmatism>1.50DC
**6–7**	All	11.0 (7.4–14.6)	0.26 (0–0.8)	7.4 (4.3–10.5)	5.4 (2.4–8.4)
	n	43	1	29	21
**12–13**	All	19.2 (16.3–22.1)	10.9 (7.4–14.4)	6.4 (4.3–8.4)	4.7 (2.8–6.6)
	n	127	72	42	31

*CIs: Confidence Intervals.*

*n = number of cases of specified refractive error.*

**Figure 1:**
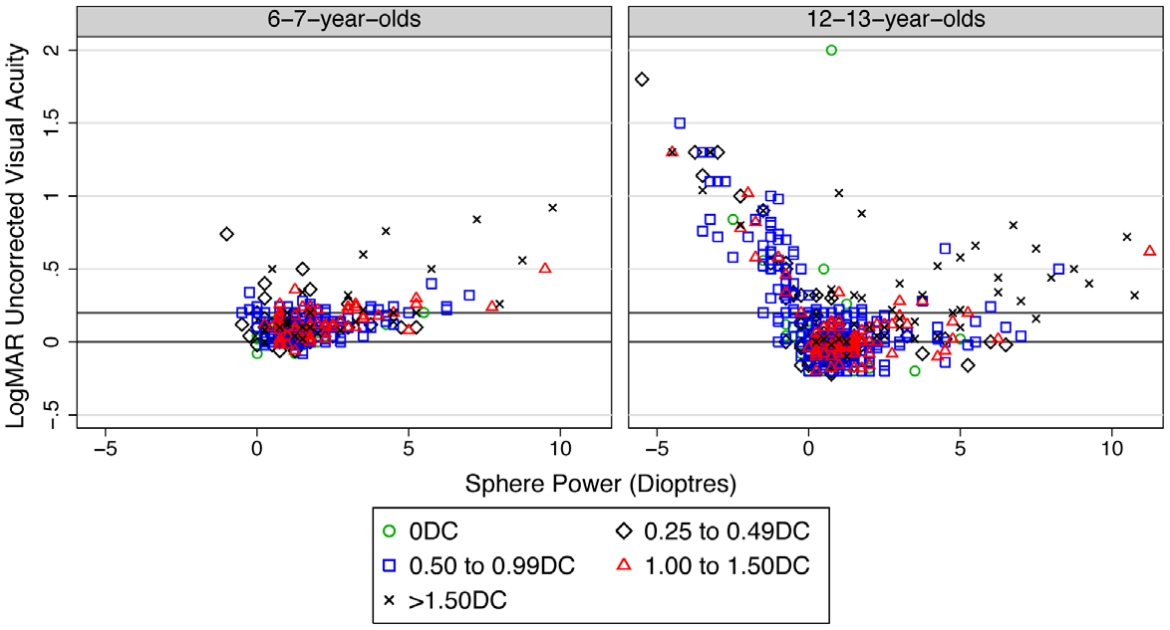
Scatterplot of uncorrected visual acuity (LogMAR) with spherical refraction for differing levels of astigmatism. The solid black lines represent 0.00 and 0.20 logMAR.

Figure 1 illustrates that the relation between uncorrected VA and the spherical component of refraction was complex. Whilst more positive spherical refraction was associated with a reduction in uncorrected VA the reduction was more pronounced when the spherical component became increasingly myopic. These data are further explored in Table 2 which confirms that myopia and astigmatism associated with myopia cause a more dramatic reduction in uncorrected VA than that occuring in hyperopia or hyperopic astigmatism. A number of children with either levels of astigmatism>1.50DC and/or significant hyperopia achieved uncorrected acuity of 0.20 logMAR or better (n = 60, 15.3% 6-7-year-olds; n = 75, 11.3% 12-13-year-olds) The sensitivity and specificity of uncorrected visual acuity measures in identifying refractive error using this criteria are presented in Table 3.

**Table 2:** Variation of uncorrected LogMAR acuity with refractive status.

	LogMAR acuity Median (IQR)	
	6-7-years	12-13-years
**Myopia**	0.74	0.70 (0.5 to 0.99)
n	1	72
**Hyperopia**	0.22 (0.14 to 0.31)	0.14 (0.02 to 0.44)
n	29	42
**No myopia/hyperopia**	0.10 (0.04 to 0.12)	−0.02 (−0.08 to 0.02)
n	361	547
**Astigmatism**	0.21 (0.12 to 0.53)	0.34 (0.20 to 0.66)
n	20	31
Myopia & astigmatism		1.04 (0.80 to 1.30)
n	0	6
Hyperopia & astigmatism	0.50 (0.20 to 0.84)	0.47 (0.33 to 0.65)
n	7	12
Astigmatism & no myopia or hyperopia	0.20 (0.10 to 0.32)	0.12 (0.10 to 0.30)
n	13	13

Definitions: myopia≤−1.00DS; hyperopia>+3.50DS; astigmatism>1.50DC.

**Table 3:** Sensitivity and specificity of an uncorrected visual acuity cut-off of poorer than 0.20logMAR to detect different refractive conditions (right eye data).

	Significant refractive error		Myopia≤−1DS		Hyperopia>+3.50DS		Astigmatism>1.50DC	
Age (years)	6–7	12–13	6–7	12–13	6–7	12–13	6–7	12–13
**Sensitivity (%)**	50	73	*	92	54	41	50	74
**Specificity (%)**	92	93		91	91	84	89	85

* n = 1.

**Figure 2:**
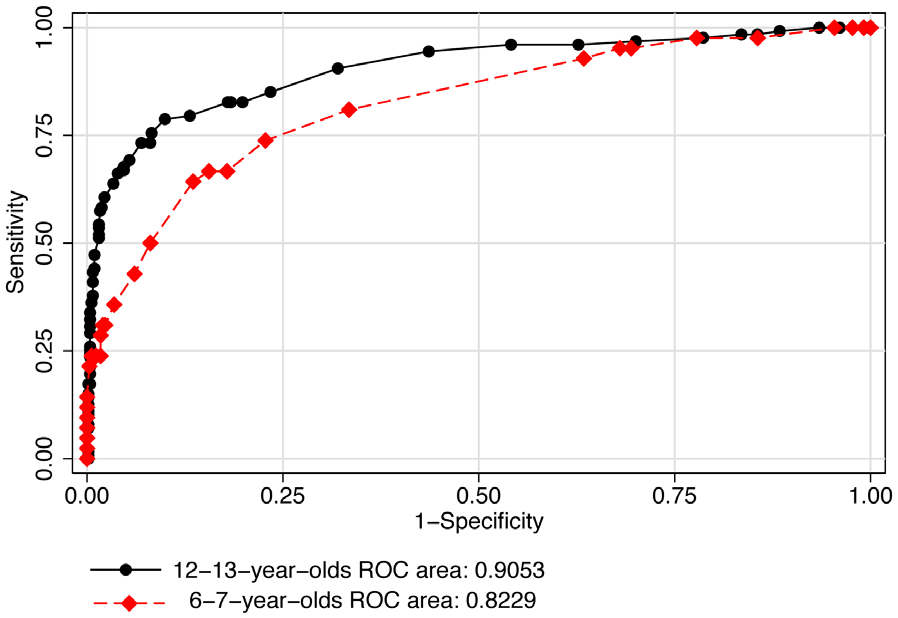
ROC curves: use of uncorrected visual acuity (LogMAR) to detect significant refractive error.

**Figure 3:**
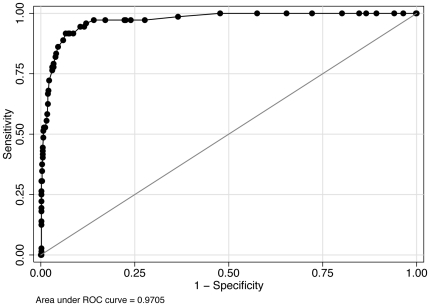
ROC curve: use of uncorrected visual acuity (LogMAR) to detect myopia in 12-13-year-olds.

**Figure 4:**
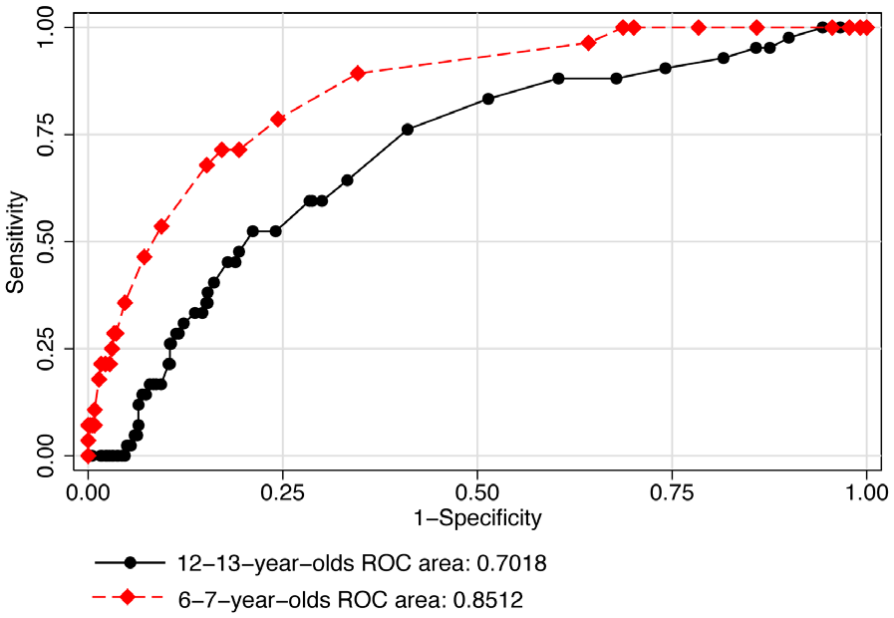
ROC curves: use of uncorrected visual acuity (LogMAR) to detect hyperopia.

**Figure 5:**
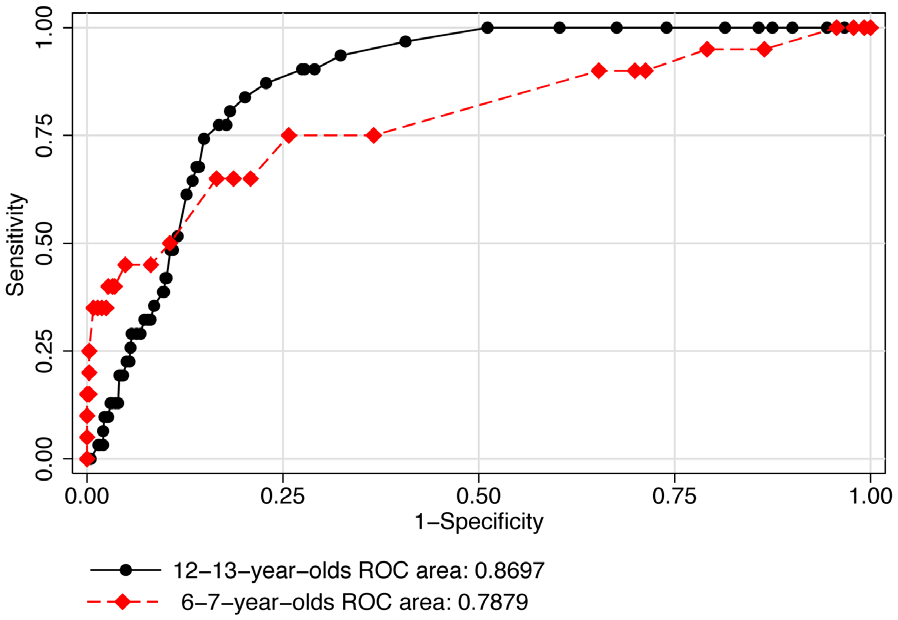
ROC curves: use of uncorrected visual acuity (LogMAR) to detect astigmatism.

Individual ROC curves were used to explore the best cut-off point for uncorrected logMAR VA (Table 4) to detect significant refractive error (Figure 2), myopia (Figure 3), hyperopia (Figure 4) and astigmatism (Figure 5).

**Table 4:** Optimal cut-off points for uncorrected visual acuity (LogMAR) to detect different refractive conditions.

	Significant refractive error		Myopia≤−1DS		Hyperopia>+3.50DS		Astigmatism>1.50DC	
Age (years)	6–7	12–13	6–7	12–13	6–7	12–13	6–7	12–13
**LogMAR Cut-off**	0.18	0.14	*	0.30	0.12	0.00	0.14	0.12
**Sensitivity (%)**	67	79		92	89	33	40	87
**Specificity (%)**	84	90		93	65	85	97	77

* n = 1.

## Discussion

Current UK vision screening guidance recommends a final universal screening of all children aged 4–5 years using logMAR distance VA. The advice is that no further vision screening interventions are currently warranted or recommended [2]. Whilst the UK National Screening Committee suggests vision screening at 4–5 years is in place to detect visual impairment including amblyopia, uncorrected refractive error and strabismus, the present study demonstrates that uncorrected logMAR acuity is poor at detecting refractive errors other than myopia. In Northern Ireland, where there is a high prevalence of hyperopia and astigmatism in childhood, screening solely on the basis of uncorrected VA will result in failure to detect many individuals with hyperopia and/or astigmatism [7], [8]. The achievement of good distance VA in the presence of refractive error does not necessarily negate the need for further investigation of visual status and management of refractive error. Hyperopia is a known risk factor for the development of strabismus and amblyopia [9]. In England, Williams et al. have shown that 34% of children with hyperopia ≥+2.00D have esotropia [19], whilst the odds ratio for esotropia is 6.4 for hyperopia from 2D to <3D, and 23.1 for hyperopia from 3D to <4D in a multi-ethnic population in the USA. In addition to visual consequences there is a growing body of evidence that uncorrected hyperopia may have a negative impact on educational attainment [12], [20] and visuocognitive and visuomotor skills [13]. Further research is required to explore these associations and indeed whether the aspiration of screening programs to identify refractive error is necessary. In the absence of such data, the UK National Screening Committee, parents, teachers and health care workers should recognise that whilst the current screening program may adequately detect amblyopia, it does not meet the diagnostic standard for a screening test for uncorrected hyperopia and astigmatism.

If vision screening programs are to identify uncorrected ametropia in childhood it may be important to employ a range of vision tests rather than rely on VA measures alone. The incorporation of a +4.00DS lens in screening programs has been proposed to help detect moderate hyperopia [12], and the public schools screening in New York City involves assessment of both distance and near acuities and the use of a +2.00DS hyperopia test [21]. However there is no firm evidence as to which tests would best support screening for ametropia.

The present study has used relatively conservative definitions of significant ametropia. The definition of myopia was based on a survey of hospital optometrists in the UK [17] and hyperopia and astigmatism on recommendations from the American Association for Pediatric Ophthalmology and Strabismus which identifies hyperopia>+3.50DS and astigmatism>1.50DC as being amblyogenic risk factors [18]. Lower levels of astigmatism (≥1DC) have also been shown to result in deficits of best corrected acuity, grating acuity, vernier acuity, contrast sensitivity and stereoacuity [22] and it has been suggested that early elementary school age children with astigmatism ≥1DC should have a trial of spectacles as they may benefit from correction [23]. The threshold for the treatment of hyperopia also remains controversial [23]. The identification of ‘significant’ hyperopia as defined by the American Association for Pediatric Ophthalmology and Strabismus does not automatically indicate that spectacle correction is necessary. However it may still be beneficial for vision screening to refer these children for a full eye examination, including assessment of visual stress symptoms, accommodation and binocular vision status and subjective refraction, in order to identify which children would benefit from correction.

This study confirms previous reports that uncorrected logMAR acuity can be used to reliably detect myopia [5], and supports the use of uncorrected visual acuity as a surrogate marker for myopia in epidemiological studies of refractive error in populations where hyperopia is not prevalent [24]. However where the prevalence of refractive error is unknown uncorrected VA gives no indication as to the type of refractive error present or to its severity.

Table 2 provides normative data for the variation of uncorrected logMAR acuity with refractive status. Whilst this table includes only right eye data, the marked reduction in uncorrected acuity with myopia compared with hyperopia and astigmatism is essentially the same regardless of which eye (right, left or better) is evaluated. Whilst the authors consider that the reduced vision associated with myopia can be attributed to a blurred retinal image due to the longer axial length of the myopic eye, this study did not evaluate the variations in an individual's ability to interpret blurred images, ocular pathology or amblyopia and these cannot be eliminated as possible causes of the reduced vision [25], [26].

In the UK, vision screening programs for children beyond primary school are no longer supported or recommended [27]. Within this context and with the knowledge that the prevalence of myopia increases with increasing age [28], the authors propose that a 0.30 logMAR screening chart (Table 4) should be placed within all secondary schools and pupils encouraged to self-refer for optometric assessment if they fail to read the letters at an appropriate distance.

### Limitations

Many childhood vision screening programs are primarily designed to detect risk factors for amblyopia rather than to detect refractive errors. As assessment of amblyopia was outside the remit of the current study, the effectiveness of VA in screening for amblyopia was not assessed.

Data collection took place in the North West of NI (Derry, Coleraine, Limavady and Ballymena) and is therefore representative of these areas specifically in terms of including urban and rural schools sampled across a range of socio-economic position. However the population of NICER will be broadly representative of NI as the Northern Irish population is relatively homogeneous.

### Conclusion

Whilst logMAR acuity of poorer than 0.20 can be used to identify myopia, its use to screen for significant refractive error results in failure to detect many children with hyperopia and/or astigmatism. Given the low levels of myopia present amongst 4-5-year-old children, the present study raises questions regarding the aspiration of vision screening programs to detect uncorrected refractive error of young children using VA measures. Further research is required to establish whether additional tests might improve detection of refractive error in this age group and whether detection and intervention is worthwhile and cost-effective.
